# Neural Substrates of Mounting Temporal Expectation

**DOI:** 10.1371/journal.pbio.1000166

**Published:** 2009-08-04

**Authors:** Jennifer T. Coull

**Affiliations:** Laboratoire de Neurobiologie de la Cognition, Pole 3C, Université de Provence, Marseille, France

## Abstract

A cognitive and neuroanatomical perspective on how timing and expectation are represented in the human brain.

When you first hear that all-too-common message that you'll have to wait—“All our operators are busy. Your call is important to us and will be answered soon.”—you're likely to accept your fate and gaze at the street scene outside your window or doodle on your notepad. But as time passes and still no one has answered, slowly you start to disengage from all other activities—you turn away from the window, you stop doodling—and, as your expectation heightens (“Surely it must be my turn by now.”), you concentrate all your attentional resources on hearing the operator's voice.

The ever-heightening temporal expectation in this everyday scenario mirrors the experimental phenomenon of the “hazard function”—the increasing conditional probability over time that an event will occur given that it has not already occurred [1],[2]. Probability is at its lowest immediately after having been put on hold, but increases monotonically with elapsing time as you become more and more convinced that, surely by now, the operator must soon reply (Figure 1A). The objectively increasing conditional probability (and, hence, the subjectively increasing sense of temporal expectation) over time relies on the predictive power of the unidirectional flow of time, or “time's arrow” [3]. Since time flows inexorably forward (at least at the psychological, macroscopic level), an event that we expect to occur, but has not yet occurred, must do so at some time in the future.

**Figure 1:**
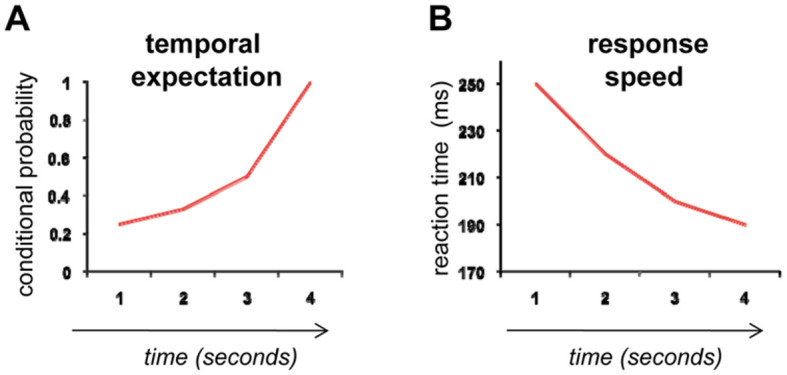
Increasing conditional probabilities over time speed responses. (A) If an event is likely to occur after one of four possible delays with equal probability, then the conditional probability that the event will occur at one of these delays evolves over time. For example, after a delay of 1 s, the probability of the event occurring is 1 in 4 (i.e., 0.25). If it does not occur at 1 s, then there are three possible delays left, now giving a 1 in 3 (i.e., 0.33) chance of occurring at the next delay. But, if it does not occur after 2 s either, there is now a 1 in 2 (i.e., 0.50) chance of it occurring at the next delay. Finally, if it has still not occurred by 3 s, then the subject can be sure that it must certainly occur (i.e., 1.0) at the final (4 s) delay. In other words, the objective probability of event occurrence combines with the predictive power of time's arrow to produce changing conditional probabilities over time. (B) As the time (or “foreperiod”) before an event occurs gets longer, so responses to that event get faster. The speeding of reaction time typically parallels increasing conditional probabilities over time, reflecting a state of increased preparedness to respond with passing time.

## Mounting Expectations Improve Performance

The ability to anticipate the timing of an event allows an organism to optimize behaviour and, thus, conserve precious resources. Experimentally, it has been known for almost a century now that as the delay (or “foreperiod”) between a warning cue and a response signal steadily increases, so too does the speed of responding to that signal [4] (Figure 1B). One long-standing theory, proposed to account for this effect, is the so-called “strategic” account [5], in which motor preparation processes are honed as a function of the increasing conditional probability of signal appearance over time. This honing process prepares the subject to respond as quickly as possible when the signal eventually appears. Although an alternative model, based on classical (Pavlovian) learning rules, has also been recently proposed [6], both models depend upon the inherently predictive nature of time's arrow to formulate their hypotheses.

Yet making use of time's arrow is not the only effective strategy for predicting event timing. Tapping your foot in time to a musical beat makes use of fixed or rhythmic, and therefore predictable, temporal sequences. Expecting an amber traffic light to turn red accesses ingrained associations between sensory cues and event timing to make temporal predictions and adjust driving behaviour accordingly. These situations are recapitulated experimentally by “temporal orienting” tasks, in which an abstract cue informs subjects that a target is likely to appear after a prespecified short delay or long delay [7],[8]. This task was originally adapted from the classic spatial orienting of attention task devised by Posner [9], in which informative cues direct subjects' attention to the right or left side of a target space. Of course, any breaches in spatial expectation are symmetrical for either side; a target appearing unexpectedly on the left is just as disruptive as one appearing unexpectedly on the right. However, because of the predictive nature of time's arrow, a target appearing later than expected is not nearly as disruptive as one appearing sooner than expected; if a target still hasn't appeared by the cued interval, it has to appear later (since it also hasn't appeared sooner), allowing expectations simply to be readjusted to the later time point, thus diluting the behavioural cost of the misleading temporal information [7].

Temporally predictable stimuli are not only detected more quickly, but perceived more accurately. For example, the timing and pitch of auditory tones are perceived more accurately when these tones occur at a regularly paced interval [10],[11]. And visual targets embedded within a rapidly presented stream of successive visual distractors are identified more readily when temporal cues inform the subject as to when the target is likely to appear within the stream [12],[13]. Temporal predictability also enhances subjective performance and can modulate more objective measures of brain activity. Single-unit electrophysiological studies in monkeys and whole-brain functional magnetic resonance imaging (fMRI) studies in humans have recently begun to identify the patterns of neural activity and regions of the brain that characterise the neural signature of mounting temporal expectation.

## How Is Temporal Expectation Represented in the Brain?

Contrary to critical opinion, fMRI does not simply provide a modern-day phrenological tool for mapping out the anatomical coordinates of distinct cognitive processes. Instead, through the use of clever experimental design and careful modelling of the data, fMRI allows us to ask not just *where* in the brain a particular process resides, but also *how* it is instantiated. In a new study published in this issue of *PLoS Biology*, Cui and colleagues [14] observed increased activation in supplementary motor area (SMA) and right superior temporal gyrus (STG) as a function of increasing foreperiod duration in a cued reaction-time task. Strikingly, they didn't simply identify areas that were increasingly activated by increasingly long foreperiods and conclude that they had identified the neural basis of mounting expectations over time (i.e., the hazard function). Instead, they formulated a series of different models that described various ways in which activity could change over time, and then calculated which of these models best fit the data.

The particular pattern of cerebral activity they observed was best fit by a cumulative hazard function, which provided an index of the integrated sum of all conditional probabilities that had been calculated over a particular foreperiod (this would correspond to the area under the curve in Figure 1A). By contrast, the worst-fitting model was one in which brain activity was hypothesised to vary linearly as a function of elapsing time, with no provision for changes in conditional probability. Taken together, these results confirm that SMA and STG activations were not simply due to increases in any nonspecific dynamic parameter that changes over time—for example, motor preparation or sustained attention—but more specifically to some evolving measure of temporal expectation that is indexed by increasing conditional probabilities. Impressively, Cui et al. [14] not only considered the possibility that their results could reflect nonspecific changes over time but also, in a series of control experiments (specifically go/no-go, countdown, and auditory analogues of their cued reaction-time task), checked that their findings were not dependent upon motor execution, motor preparation, or sensory modality, respectively.

Intriguingly, the SMA and STG effects were not observed *during* the foreperiod, as might be expected. Previous single-cell electrophysiological studies in monkeys have measured neural firing patterns on a moment-to-moment basis during the foreperiod, and showed progressive changes in firing as the foreperiod evolved [15]–[17]. Consequently, Cui et al. [14] sought to measure similar changes in human subjects using fMRI. But, despite several attempts to model progressively increasing (or decreasing) changes in neural activity during the foreperiod itself, they failed to find any brain area where activity consistently evolved as a function of foreperiod length. Instead, they observed two discrete bursts of activity in SMA and STG upon presentation of the response signal *after* the foreperiod had ended. Nevertheless, the relative amplitude of these bursts of activity varied as a function of the length of the preceding foreperiod—the longer the foreperiod, the greater the burst of activity. This is consistent with the aforementioned effect on an integrated or cumulative hazard function: the SMA and STG results do not necessarily index the dynamic evolution of the hazard function over time (i.e., during the foreperiod) but, rather, the end-result of the computation (i.e., when the appearance of the response signal terminates the foreperiod, thus indicating its final length and, thus, its associated cumulative probability). What can this unexpected result tell us about the representation of time in the brain?

The most widely cited psychological model of time is the pacemaker-accumulator model [18]. In this model, a sensory signal (e.g., the onset of a stimulus to be timed) triggers an accumulator to begin counting pulses that are emitted by an internal pacemaker. The accumulated pulse tally is then passed into working memory for comparison with a previously stored pulse tally. Could the cumulative hazard function in SMA and STG [14] correspond to this accumulation of pulses? The results of previous fMRI studies showing preferential activity in SMA for long rather than short durations [19],[20] make this an appealing proposition. However, if this were true, Cui et al.'s [14] data would have been equally well described by the simplest model in which brain activity evolves linearly with time. In fact, they showed that the inclusion of an additional factor, the hazard function, explained the data much more completely. The differential explanatory power of the hazard function versus linear models indicates that these cortical effects represent something more akin to the updating of temporal expectations as a function of evolving conditional probabilities, rather than simple estimation of time-in-passing.

Of course, one alternative possibility is that it may be impossible to estimate time-in-passing *without* making use of the inherent predictability of time's arrow, thus explaining why hazard function models fit the data so much better than simple linear ones. If this were so, psychological models of time would have to be reworked so that time estimation is not just a simple case of blindly accumulating pulses as they occur, with no end-point in sight. Instead, the requirement to estimate duration would simultaneously invoke a temporal context, such that subjects would be timing while keeping in mind the likely moment of stimulus offset. In fact, including an element of temporal expectation in models of time estimation may actually reflect a more ecologically valid conceptualisation of how we time events in the real world. We very often judge the duration of an event based on an expectation of how long it normally lasts (“The bus should have been along by now.”), rather than timing the event chronometrically (“I've been waiting at the bus stop now for one minute…two minutes…three minutes…”).

## A Functionally Distributed Anatomical Network for Temporal Expectation

**Figure 2:**
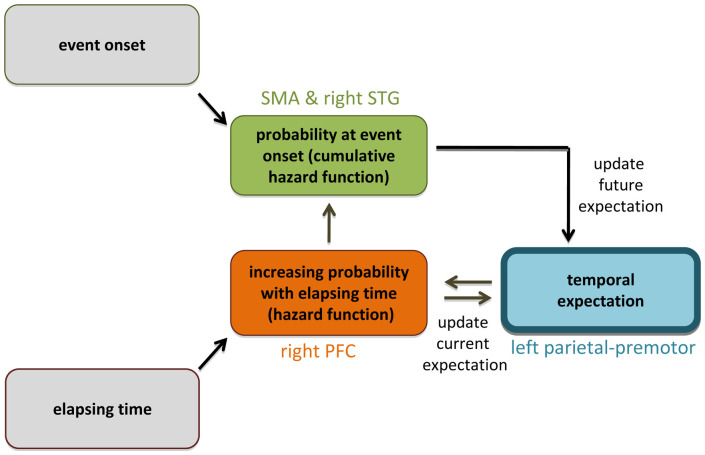
Temporal expectation in the brain. Fixed temporal expectations of when a visual event is likely to occur are underpinned by activity in left premotor and parietal areas [7],[26]. However, if the event has still not appeared by the expected delay, the right prefrontal cortex (PFC) [21]–[25] makes use of neural indices of elapsed time (represented in functionally specialized regions of the brain e.g., in visual cortex for visual events [15]) to update current temporal expectations (i.e., the hazard function). Once the event occurs, an integrated sum of the probability that the event would have occurred at that time (i.e., the cumulative hazard function) is represented by the magnitude of activity in SMA and right STG [14], and allows expectations about the onset time of future events to be updated.

Cui et al. [14] indexed the hazard function by parametrically modelling fMRI data as a function of foreperiod length. Previous fMRI studies have indexed the hazard function by contrasting trials in which temporal expectation increases as a function of time versus those in which temporal expectation remains fixed throughout trial duration. In a temporal orienting task, trials that afforded time for temporal expectations to be updated during the foreperiod engaged right prefrontal and premotor activity [21]. This result was later supported by an impressive series of methodologically diverse neuroscientific investigations of cued reaction-time tasks [22]–[25], showing that right prefrontal cortex was necessarily implicated in the monitoring or updating of changing conditional probabilities over time. In these studies, the predictive nature of time-in-passing was used to increase response speed, and this behavioural benefit was shown to be dependent upon right prefrontal activation. On the other hand, when *fixed* temporal expectations have been used to optimise response speed (e.g., by temporally informative cues or temporally predictable sequences), activation of left-lateralised parietal and ventral premotor circuits is observed [7],[26]. Together, these results suggest that temporal expectations are established in left parietal–premotor action circuits but are monitored, and potentially updated on-line, as a function of time-in-passing, by the right prefrontal cortex (Figure 2). Cui et al.'s [14] new findings complement these data by showing that, once the expected signal or event occurs, SMA and right STG provide an integrated tally of how that expectation evolved over time.

The functional purpose of this integrated tally is still unknown, however. Cui et al. [14] suggest it could act as a signal of prediction error, perhaps providing feedback that might be used to improve the accuracy of future temporal expectations (Figure 2). Alternatively, as suggested above, it could potentially reflect an index of accumulated time, if one accepts that time is measured not only as a function of elapsed time but also as a function of expected time. Although these proposals are entirely speculative, they provide substantial fodder for future investigation. Do we ever actually time an event without anticipating when it will end? Electroencepholographic (EEG) studies of duration estimation in humans have shown evidence of climbing neural activity in medial frontal electrodes (i.e., in the region of SMA) that was synchronised to the expected, not the objective, moment of stimulus offset [27]. Perhaps magnetoencephalography (MEG), with its superior temporal and spatial resolution, would provide a more fruitful technique in the future for identifying regions of climbing neural activity during the foreperiod itself. Such experiments may help shed some light on the functional mechanism of timing. Can we identify differential patterns of neural activity that are best characterised as a linear function of time versus patterns of activity that vary in line with increasing conditional probabilities? Are some brain areas consistently activated by timing regardless of task context (centralised representation), or is time represented in functionally specialised regions of the brain that differ depending on the sensory or motor nature of the task (local representation)? Is there a critical window within which timing passes from being locally represented to being centrally represented? Recent developments in the spatial and temporal resolution of cognitive neuroscience techniques (e.g., fast event-related fMRI, MEG) will help answer these, and many other, questions of time.

## Abbreviations

fMRI: functional magnetic resonance imaging
MEG: magnetoencephalography
STG: superior temporal gyrus
SMA: supplementary motor area
