# Correction: Neural Substrates of Mounting Temporal Expectation

**DOI:** 10.1371/journal.pbio.3001118

**Published:** 2021-02-10

**Authors:** The PLOS Biology Staff

## Notice of Republication

This article was republished on 29 January 2021, to correct the online version of the article which displayed an incorrect Fig 1. The PDF version of Fig 1 was correct. The publisher apologizes for the errors.
